# CEO Relational Leadership and Product Innovation Performance: The Roles of TMT Behavior and Characteristics

**DOI:** 10.3389/fpsyg.2022.874105

**Published:** 2022-04-27

**Authors:** Yimin Wang, Qianhong Su, Wei Sun

**Affiliations:** School of Management, Shandong University, Jinan, China

**Keywords:** CEO relational leadership, product innovation performance, TMT voice behavior, TMT education, TMT age

## Abstract

CEO leadership is considered a critical antecedent of product innovation performance, but the relational aspect of leadership has been largely neglected in this area. Drawing on upper echelons theory and relational leadership literature, this study explores *whether*, *how*, and *when* CEO relational leadership influences product innovation performance. Specifically, we analyze the underlying mechanism of TMT (top management team) voice behavior and two boundary conditions—TMT educational level and TMT age. Based on multi-source and multi-wave data on 105 Chinese firms, this study finds that CEO relational leadership plays an important role in promoting product innovation performance through the intervening mechanism of TMT voice behavior. Furthermore, the positive relationship between CEO relational leadership and TMT voice behavior is stronger in TMTs with higher educational level and lower age. This study contributes to the existing literature by empirically examining the under-investigated relationship between CEO relational leadership and product innovation performance, and by disentangling the underlying mechanism and boundary conditions.

## Introduction

A wealth of research has suggested that leadership, especially at a strategic level, is a vital predictor of product innovation (Felekoglu and Moultrie, 2014; Hughes et al., 2018) because strategic leaders and their leadership behavior in a firm are not only responsible for strategic decisions on product innovation, but also affect individuals’ innovative behavior by shaping organizational structure and innovative culture (Hambrick, 2007; Cortes and Herrmann, 2021). Prior research has examined the effects of various leadership traits and behaviors of CEOs on firm innovation, including traditional ones such as CEO transformational leadership (Jung et al., 2008; Cortes and Herrmann, 2020), CEO transactional leadership (Prasad and Junni, 2016), CEO servant leadership (Ruiz-Palomino et al., 2019), and emerging ones such as creative CEO leadership (Makri and Scandura, 2010) and CEOs’ visionary innovation leadership (Caridi-Zahavi et al., 2016).

These leadership studies have made significant contributions to our understanding of how CEO leadership affects innovation. However, the relational aspect of leadership has been largely neglected in the context of product innovation. Rather than focusing on the individual traits or behaviors of leaders in the appointed position, relational leadership pays special attention to interpersonal relationships and relational process to capture what actually happens in the “space between” leaders and followers, and how leaders interact with others (Fletcher, 2004; Uhl-Bien, 2006). It “validates the importance of leadership oriented to enhancing relationships among individuals or organizations (Quick, 2014: 542).” This is highly relevant to new product development activities, as product innovation is a “collective achievement” that involves intensive interaction and collaboration among individuals and across functions to successfully bring a new idea to production and to the market (Van de Ven, 1986; De Luca and Atuahene-Gima, 2007). Notwithstanding the potential benefits that relational leadership brings to innovation, little empirical research has been conducted on whether and how relational leadership affects product innovation performance.

To address these issues, this study, based on upper echelons theory and the relational leadership literature, explores *whether*, *how*, and *when* CEO relational leadership affects product innovation performance. According to upper echelons theory, the CEO has a great impact on firm outcomes (Hambrick and Mason, 1984), and research has demonstrated that CEO leadership can promote firm innovation (Ling et al., 2008; Zhang et al., 2015). By encouraging individuals to engage in relational interaction and collaboration, relational CEOs may help speed up information exchange and knowledge sharing, reduce conflict, and develop interpersonal relationships and trust (Komives et al., 1998; Carmeli et al., 2012), which are all indispensable for successful product innovation.

To elaborate on how this process unfolds, we introduce TMT voice behavior as a mediating mechanism. Upper echelons research has always been criticized for lacking the exploration of underlying process through which CEO characteristics affect firm outcomes (Carpenter et al., 2004). The latest reviews on upper echelons theory state that TMT process or TMT dynamics can act as the key underlying mechanisms to unpack the “black box” between CEO characteristics and distal firm outcomes (Abatecola and Cristofaro, 2020; Neely Lovelace et al., 2020). TMT voice behavior—the extent to which TMT members express themselves to challenges the *status quo* and bring about constructive changes (Walumbwa et al., 2012; Frazier and Bowler, 2015)—may damage or upset interpersonal relationships. It represents the interpersonal dynamics within the TMT, which is largely shaped by CEOs and affects firm strategies and outcomes (Carpenter et al., 2004; Tang et al., 2021). Furthermore, voice behavior has been acknowledged to have great benefits for innovation because it challenges obsolete practices, brings about novel ideas, and improves efficiency (LePine and Van Dyne, 1998; Detert et al., 2013). Given the strategic positions of TMT members, their voices can have a direct and significant effect on the firm’s strategic decisions and performance related to product innovation.

In addition, this study explores the boundary conditions to extend the understanding of when CEO relational leadership may lead to TMT voice behavior. How top managers interpret and respond to external stimuli depends on their values and cognition reflected by demographic characteristics, such as education and age (Hambrick and Mason, 1984). When relational CEOs signal that they are willing to listen to suggestions and embrace new ideas, TMT members with different educational levels and ages may respond differently. Prior research has demonstrated that TMT members with higher educational achievement and lower age are more likely to absorb new ideas and accept change and innovation (Bantel and Jackson, 1989; Hitt and Tyler, 1991; Smith et al., 2005). Thus, TMT members with higher educational attainment and lower age are more likely to be motivated by relational CEOs to put forward their unique ideas and perspectives, which may then promote product innovation performance.

We contribute to the existing literature in followings ways. First, by examining whether, how, and when CEO relational leadership can promote product innovation performance, this study contributes to the literature on CEO leadership and firm innovation from the relational perspective of leadership. Although CEO leadership has been widely examined in the context of innovation, the relational aspect has been ignored. We argue that the relational aspect of CEO leadership provides new insights on how a CEO’s leadership style promotes firm innovation. Second, by introducing the mediating role of TMT voice behavior, this study complements research on how CEO leadership translates to firm innovation and enriches the CEO–TMT interface literature. Third, by identifying two conditional factors, this study also contributes to the understanding of when CEO leadership facilitates TMT or employee voice behavior. Although prior research has noted the positive effect of CEO leadership and employee voice behavior, it has ignored the boundary conditions. Finally, this study enriches the literature on relational leadership by empirically examining its outcomes.

## Theoretical Background and Hypotheses

### Theoretical Background

As the most powerful person in a firm, the CEO has an outsize effect on firm outcomes; this has been widely examined from various angles, including CEOs’ demographic characteristics, cognitive attributes, and leadership behavior (Hambrick and Mason, 1984; Liu et al., 2018). Prior research has explored various types of CEO leadership and their effects on firm innovation. For example, scholars have found that CEO transformational leadership is positively related to product innovation by motivating employees’ innovative behavior and intrinsic motivation (Jung et al., 2008; Chen et al., 2014; Cortes and Herrmann, 2020). Ruiz-Palomino et al. (2019) found that CEO servant leadership can promote hotel innovativeness by being humble and serving employees. Caridi-Zahavi et al. (2016) specified an emerging leadership—CEO visionary innovation leadership—and showed that CEOs can motivate knowledge integration and firm innovation by creating and conveying their visions for innovation.

Although the existing research has made significant contributions in this area, most leadership theories view leadership as a property of CEOs and place their attributes or behaviors at the center of our understanding of leadership (McCauley and Palus, 2021). These studies have been criticized for paying little attention to the relational process of leadership (Uhl-Bien, 2006; Jian, 2022). Moreover, some scholars of product innovation have called for more research on the “human side” of top managers’ involvement, that is, how top managers interact with others (Brenton and Levin, 2012; Felekoglu and Moultrie, 2014). The emphasis on the “human side” in product innovation research is in line with the “relationality movement” in leadership development, which has gained increasing attention from scholars (Uhl-Bien and Ospina, 2012; McCauley and Palus, 2021).

According to Uhl-Bien (2006), relational leadership is “a social influence process through which emergent coordination (e.g., evolving social order) and change (e.g., new approaches, values, attitudes, behaviors, ideologies) are constructed and produced” (p. 668). Based on entity perspective and relational perspective, it not only includes relationship quality but also relational processes among people (Hunt and Dodge, 2000; Uhl-Bien, 2006). Particularly, the relational process and its dynamics, in the form of daily interaction and dialog, are at the core of relational leadership (Cunliffe and Eriksen, 2011; Endres and Weibler, 2017). By focusing on the relational interaction and process, relational leadership moves beyond the traits or behaviors of leadership (Reitz, 2015). Komives et al. (1998) characterize relational leadership using five elements: purpose, inclusiveness, ethic, empowerment, and process orientation. Purpose means that relational leaders have the ability to develop shared goals and vision. Being inclusive involves understanding and respecting differences and diversity. Being process-oriented means that relational leaders emphasize the interaction process and encourage cooperation and communication. Relational leaders are also ethical and empowering (i.e., willing to share power).

Although some traditional leadership theories (e.g., transformational leadership) and relational leadership all emphasize vision, respect, and trust, transformational leadership, as one of the entity studies, focuses on the transformational characteristics of persons in leadership positions and their asymmetry effects on subordinates (Van Knippenberg and Sitkin, 2013). As explained by Jian (2022), these studies “fall short in offering an adequate account of the constitutive role of communication and discourse” (p. 2), whereas relational leadership places greater emphasis on interactions and communication processes through which collective learning and mutual influence occur (Fletcher, 2004). Shared vision and trust are not conveyed from a leadership position but co-created through the process of relating to and interacting with others. Relational leaders pay special attention to personal interaction and mundane conversations rather than focusing only on control and authority (Denis et al., 2012; Endres and Weibler, 2017).

These relational practices are especially important for product innovation in the era of rapid technological change and intensifying competition. Product innovation is not only a technological process but also a social activity that involves intensive interaction and collaboration among workers (Van de Ven, 1986). When organization members are encouraged to engage in daily communication and interaction with each other, knowledge sharing and new ideas that are critical for product innovation may also occur (Anderson et al., 2014). During the interaction, collective learning and shared understanding about product innovation can also be achieved (Fletcher, 2004; Hosking, 2007). Despite these potential benefits, the relational dimension of leadership has been largely ignored in the context of product innovation. Therefore, it is necessary to explore the relationship between CEO relational leadership and product innovation performance.

**FIGURE 1:**
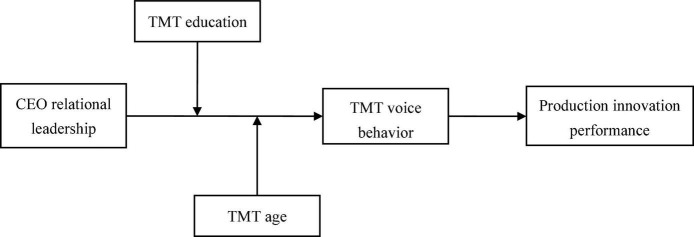
Theoretical model.

In this study, we use upper echelons theory as the theoretical framework. Upper echelon theory states that top managers’ values and cognition have a great impact on their perception of the environment, which, in turn, affects firms’ strategic decisions and performance (Hambrick and Mason, 1984). Researchers have applied upper echelons theory to analyze the effects of different types of CEO leadership on firm outcomes (Zhang et al., 2015; Mallen Broch et al., 2020). However, upper echelons research has often been criticized for not exploring the “black box” between CEO characteristics and distal firm outcomes (Neely Lovelace et al., 2020). To unpack the underlying mechanism, researchers have focused on the mediating role of TMT dynamics, such as TMT potency (Zhang et al., 2015) and TMT integration (Ling et al., 2008). In this study, we introduce TMT voice behavior as one aspect of TMT dynamics to explain the underlying mechanism. The theoretical model is presented in Figure 1.

### CEO Relational Leadership and TMT Voice Behavior

Based on Walumbwa et al. (2012), TMT voice behavior can be defined as the extent to which TMT members express concerns, make constructive suggestions, and propose innovative ideas to their CEO with the aim of achieving positive change (LePine and Van Dyne, 1998; Detert and Burris, 2007). Although extant studies have not investigated the effect of CEO relational leadership on TMT voice behavior, an increasing number of analyses have pointed out that relational leadership is inherently linked to linguistic practices and open dialog (Cunliffe and Eriksen, 2011; Reitz, 2015), which provide the opportunity for TMT members to express their concerns and opinions.

Specifically, relational leadership emerges from the relational interaction (Fletcher, 2004; Endres and Weibler, 2017). Dialog, which is a two-way communication characterized by the willingness to listen to others and giving respond, is one of the main forms of relational interaction (Cunliffe and Eriksen, 2011). Being engaged in daily dialog by relational CEOs, TMT members have the opportunity to state their concerns about the firm’s operation and provide constructive input (Fletcher, 2004). Relational CEOs also give feedback and convey important information to TMT members to strengthen their feeling of being involved and valued in the firm, which in turn increases their voice behavior (Binyamin and Brender-Ilan, 2018). This rich interaction also increases TMT members’ perception of the CEO’s openness and approachability, which is positively related to subordinates’ voice behavior (Detert and Burris, 2007).

Furthermore, relational leadership emphasizes relationship quality and mutual understanding among members, which may free TMT members from the fear of voicing their opinions or concerns. Although voice behavior is intended to benefit the organization, it may also embarrass others, upset interpersonal relationships, and result in punishment (Detert and Burris, 2007; Liang et al., 2012). CEO relational leadership can foster a climate of trust among TMT members by building positive relationships and facilitating collaboration among them (Carmeli et al., 2012). A supportive relationship between leaders and their subordinates frees the latter from fear of being punished for challenging the *status quo* (Morrison, 2011; Cai et al., 2019).

Relational leaders also respect the differences among their subordinates; they are open to different views and willing to explore new possibilities (Cunliffe and Eriksen, 2011). They tend to believe that every individual can make a difference and encourage their subordinates to speak up and make contributions to the firm (Komives et al., 1998; Hosking, 2007).

Based on the arguments above, we propose the following hypothesis:

*Hypothesis 1: CEO relational leadership is positively related to TMT voice behavior.*

### The Mediating Role of TMT Voice Behavior

Successful product innovation involves two stages: coming up with constructive ideas in the idea generation stage, and successfully guide a new idea to production and finally to the market in the implementation stage (Van de Ven, 1986; Anderson et al., 2014). We argue that TMT voice behavior is crucial to these processes.

First, TMT voice behavior promotes the generation of creative ideas, whether they are a modification of old concepts or entirely new ones (Van de Ven, 1986; LePine and Van Dyne, 1998; Anderson et al., 2014). Literature on voice behavior has argued that new ideas are often embedded in voice behavior (Guzman and Espejo, 2019) because voice behavior emphasizes the expression of suggestions to bring positive change and challenge the *status quo* (Van Dyne et al., 2003; Detert et al., 2013). In addition, TMT members voicing distinct opinions or ideas is more likely to stimulate an intensive discussion where information exchange occurs and new insights are gained (Chou and Walker-Price, 2018; Guzman and Espejo, 2019). For instance, Liang et al. (2019) found that team member voice is positively related to team innovation because it involves the integration of others’ knowledge in the production of new ideas and solutions.

Second, TMT voice behavior bridges ideas and the implementation of innovation; it also improves the effectiveness of new product development (Rank et al., 2004; Rasheed et al., 2017). The implementation of innovation is a process of learning that involves the adoption of new procedures and coordination across different units (Montoya-Weiss and Calantone, 1994). TMT members can facilitate the adoption of new routines by expressing their concerns and sharing their ideas on how to implement new practices in their respective departments (Edmondson, 2003). Speaking out about problems and flaws spotted in new products can also reduce the cost of mistakes and improve the effectiveness of new product development (Morrison and Milliken, 2000; Liang et al., 2019). In addition, initiating a new project inevitably results in conflict and resistance from different interest groups (Kim et al., 2010). TMT voice behavior can address such conflict through social interaction and relationship-building and facilitate the successful implementation of innovation (Nguyen et al., 2017).

Finally, the targets of TMT voice behavior are CEOs or other TMT members who occupy dominant positions in the organization and have control over resources (Hambrick, 1994). When TMT members voice their innovative ideas and concerns to the CEO, they may get an immediate response and thus take quick action to develop new products (Detert et al., 2013). As competition becomes fiercer, the speed of new product development is increasingly important for firms to sustain competitive advantages. TMT voice behavior can lead to rapid product development and thus to seizing of market opportunities.

Based on the arguments above, we propose the following hypothesis:

*Hypothesis 2: TMT voice behavior is positively related to product innovation performance.*

Although upper echelons theory predicts that top executives’ characteristics are reflected in firm strategies and outcomes (Hambrick and Mason, 1984), CEOs as leaders of TMTs have “dominating influence” on TMT functions and shape their characteristics (Hambrick, 1994; Liu et al., 2018). The literature has also shown that the CEO’s effect on firm outcomes is transmitted by TMT dynamics (Ling et al., 2008; Carmeli et al., 2012; Zhang et al., 2015). TMT voice behavior can act as an interpersonal dynamic within TMTs (Tang et al., 2021) because voicing opinions or suggestions not only involves interpersonal communication, but also engenders conflicts or embarrassment by challenging others which may strain interpersonal relationships and alter the interpersonal dynamics within TMTs (Detert and Burris, 2007). Therefore, based on H1 and H2 and the arguments above, we argue that the effect of CEO relational leadership on product innovation is mediated by TMT voice behavior. Relational CEOs who develop good relationships with TMTs and engage them in dynamic interactions decrease the perceived risk of speaking up and increase the opportunity for TMTs to express their suggestions, which in turn promotes the generation of new ideas that advance product innovation performance.

*Hypothesis 3: TMT voice behavior mediates the positive relationship between CEO relational leadership and product innovation performance.*

### The Moderating Role of TMT Characteristics

The effect of CEO relational leadership on TMT voice behavior may not be the same for all TMT members. According to upper echelons theory, top managers with different values and cognition reflected by demographic characteristics (such as education and age) react differently to external stimuli, and their reaction affects strategic choices and decisions (Hambrick and Mason, 1984). When relational CEOs signal their willingness to embrace suggestions and ideas, some individuals respond actively, while others may keep silent. We consider two prominent demographic characteristics—TMT educational level and TMT age—as moderating variables in the relationship between CEO relational leadership and product innovation performance.

#### TMT Education

Educational level is an indicator of an individual’s knowledge stock, skills, and cognitive abilities (Hambrick and Mason, 1984; Bantel and Jackson, 1989). Previous research has provided evidence that a higher educational level is associated with higher cognitive complexity, the ability to process information, the absorption of new ideas, and the acceptance of change and innovation (Hitt and Tyler, 1991; Wiersema and Bantel, 1992; Smith et al., 2005). Cognitive complexity represents the ability to process complicated problem and make decisions under ambiguity (Hitt and Tyler, 1991). Being open to new ideas and change also makes these educated people skillful at acquiring new knowledge and sensitive to new opportunities (Capelleras et al., 2019; Nuscheler et al., 2019). Relational CEOs are more likely to seek advice from highly educated executives and create a supportive climate for them to express themselves freely.

Furthermore, highly educated executives who have a higher aspiration for business growth and will devote more commitment to the firm (Capelleras et al., 2019). They are more likely to be motivated by relational CEOs to express their ideas and suggestions based on their professional knowledge. Through frequent interaction and dialog, relational CEOs also let them know they are trusted and valued, and increase their willingness to point out problems and voice suggestions (Cai et al., 2019). Based on the arguments above, we posit that when TMTs have a higher educational level, the positive relationship between CEO relational leadership and TMT voice behavior is stronger.

*Hypothesis 4a: The positive relationship between CEO relational leadership and TMT voice behavior is stronger when the average educational level of TMTs is higher.*

#### TMT Age

TMT average age reflects members’ general physical and psychological resources and is strongly linked to work-related abilities (von Bonsdorff et al., 2018). Older executives suffer both a reduction in physical resources and loss of cognitive ones (Wang and Shultz, 2010). They may need more time to learn new skills, which often makes them anxious. Therefore, they tend to insist on existing procedures and routines and resist the new ideas and change (Stirpe et al., 2018). Moreover, executives approaching retirement may have a narrow career horizon that emphasizes their career and financial security and lowers their commitment to work (Herrmann and Datta, 2005; Wang and Shultz, 2010). It is difficult for relational CEOs to change their attitude and engage them in substantial dialog.

In contrast, younger executives are full of energy and eager to learn new skills; they tend to come up with constructive ideas and grasp new opportunities (Heyden et al., 2017). They are more likely to consider riskier strategies, like R&D spending, innovation, and corporate change (Wiersema and Bantel, 1992; Barker, and Mueller, 2002) and to put emphasis on career development prospects rather than career security (Barker, and Mueller, 2002; Herrmann and Datta, 2005). Furthermore, younger TMT members are keen to engage in management and participation-enhancing practices to acquire experience and expert knowledge (Hitt and Tyler, 1991; Stirpe et al., 2018). It is easier for relational CEOs to engage them in dialog and create a climate for them to voice their suggestions and ideas. In consequence, when TMT members are younger, the positive relationship between CEO relational leadership and TMT voice behavior becomes stronger.

*Hypothesis 4b: The positive relationship between CEO relational leadership and TMT voice behavior is weaker when the average age of TMTs is higher.*

## Methods

### Sample and Procedure

The sample consisted of 105 small and medium-sized enterprises (SMEs) located in eastern China, most of them (92.4%) privately held. Small and medium-sized enterprises exert fewer constraints on CEO and TMT discretionary behavior (Hambrick and Finkelstein, 1987), their CEOs and TMTs play a more direct role in influencing firm strategies and outcomes. Hence, we collected data in SMEs with the support of local government agencies and personal network. Initially, we contacted CEOs in SMEs through our personal network, and then asked them to recommend other CEOs. Furthermore, one of the CEOs helped us access the Small and Medium Business Service Association, which provided us with additional contact information for more SMEs. With the support of the government agency, we got access to another 150 SMEs. A total of 200 SMEs (with 50 SMEs by personal network) would participate in our research.

To reduce common method biases (Podsakoff et al., 2003), we conducted a multi-source and multi-wave method to collect the survey data during 2017–2018. In the first wave (conducted in August 2017), research assistants explained the purpose of the research project and promised to keep the responses confidential. Then questionnaires were mailed to the target firms along with prepaid envelopes. TMT members were asked to rate their CEO’s relational leadership and their own voice behavior, and to provide background information on themselves (age, education, gender, tenure, and how long they had worked with their CEO). We received 182 completed surveys, with a response rate of 91%. After matching, 168 sets of questionnaires could be used.

In the second wave (one year after the first), we asked the CEOs of the 168 firms to assess their firm’s product innovation performance and to provide information on the firm and on their own demographics. A total of 125 firms replied to this second survey. After deleting incomplete questionnaires, 105 sets of usable and matched questionnaires were retained for hypothesis testing.

The final sample thus included 105 firms, 70.5% of which were in the high-technology industry, 28.5% in manufacturing, and 1% in services and other industries. Among the final sample of 105 CEOs (84 males and 21 females), the average age was 44.36 years (*SD* = 7.97) and the average tenure was 12.65 years (*SD* = 7.19). Among the TMT members, 62.9% had a bachelor degree or higher, the average age was 38.93 years (*SD* = 6.08), the average team tenure was 8.04 years (*SD* = 4.66), and the average duration of working with their leader was 6.38 years (*SD* = 3.45).

### Measures

We translated the survey from English to Chinese following Brislin (1980) back translation procedure to ensure equivalence of meaning. Unless indicated otherwise, all ratings were made using a five-point Likert scale (from 1 = “Strongly disagree” to 5 = “Strongly agree”).

#### CEO Relational Leadership

We measured relational leadership using a seven-item scale from Hernandez et al. (2014) to measure observable behavioral tendencies as perceived by TMT members. The seven items were “Our CEO cares about our individual priorities and interests,” “Our CEO is sensitive to our needs,” “Our CEO displays concern for us,” “Our CEO deals fairly with us,” “Our CEO shows respect for people regardless of their level in the organization,” “Our CEO is unbiased in his/her decisions” and “Our CEO makes an effort to seek out others’ opinions on important issues.” Cronbach’s reliability coefficient alpha value was 0.95, indicating acceptable reliability. Statistical checks showed high inter-rater agreement between the TMT members within each firm (average Rwg = 0.95), with ICC (1) and ICC (2) values of 0.13 and 0.42, respectively, so the responses were aggregated and used as the measure of relational leadership.

#### TMT Voice Behavior

We adopted a five-item scale from LePine and Van Dyne (1998); Liang et al. (2012) to measure voice behavior. Items included “I proactively develop and make suggestions for issues that may influence our firm,” “I proactively suggest new projects which are beneficial to our firm,” “I raise suggestions to improve my firm’s working procedure,” “I proactively voice constructive suggestions that help our firm reach its goals” and “I make constructive suggestions to improve the firm’s operation.” The Cronbach’s alpha of this measure was 0.91, indicating good reliability. TMT voice behavior was a firm-level variable. The TMT members’ responses showed high inter-rater agreement among TMT members within the same firm (average Rwg = 0.94), with ICC (1) and ICC (2) values of 0.31 and 0.63, respectively. The responses were therefore aggregated and used as the measure of TMT voice behavior.

#### Product Innovation Performance

Product innovation performance was rated on a five-item scale developed by De Luca and Atuahene-Gima (2007), by asking CEOs to evaluate the extent of their firm’s new product and service development in market share, sales, return on assets, return on investment, and profitability. Items included “Our firm’s new product/service development has achieved a market share relative to the firm’s stated objectives in the past year,” “Our firm’s new product/service development has achieved the return on assets relative to stated objectives in the past year,” “Our firm’s new product/service development has achieved the return on investment related to stated objectives in the past year,” “Our firm’s new product/service development has achieved the sales relative to stated objectives in the past year,” and “Our firm’s new product/service development has achieved the profitability relative to stated objectives in the past year.” Cronbach’s alpha was 0.94.

#### TMT Demographic Characteristics

We selected average educational level (TMT members’ education) and average age (TMT members’ age) as demographic characteristics. As education is a categorical variable, we measured it after encoding the level (0 = junior high school or below; 1 = high school; 2 = junior college; 3 = undergraduate; 4 = master’s degree or higher).

#### Control Variables

We controlled for CEO and TMT characteristics and firm characteristics. For CEO and TMT characteristics, we included basic demographic information commonly used as controls in related studies (Tang et al., 2020), including CEO’s age, CEO’s gender (0 = male, 1 = female), CEO’s tenure (in years), TMT members’ tenure and TMT members’ working time with their leader (both in years). At the firm level, we included whether the firm was privately held (0 = no, 1 = yes) and the industry in which it is active (1 = high-technology, 2 = manufacturing, 3 = services and other).

## Results

### Testing the Measurement Model

Table 1 displays the means, standard deviations, and correlations of the focal variables. All values of Cronbach’s alpha were above the minimum acceptable threshold of 0.70, confirming the reliability of the established scales. The correlations between the focal variables were statistically significant and in the expected direction. Relational leadership was positively and significantly correlated with TMT voice behavior (*r* = 0.44, *p* < 0.001). TMT voice behavior was positively and significantly correlated with product innovation performance (*r* = 0.35, *p* < 0.001). These results provide initial support for our hypotheses. As shown in Table 1, all values of the variance inflation factor (VIF)—an indicator of multicollinearity—are less than 10, as recommended by Vittinghoff et al. (2012), so multicollinearity was not present in this model.

**TABLE 1:** Descriptive statistics.

Variables	VIF	1	2	3	4	5	6	7	8	9	10	11	12
(1) Relational leadership	1.30	*(0.95)*											
(2) TMT voice behavior	1.54	0.44***	*(0.91)*										
(3) TMT education	1.28	0.17	0.23*	1									
(4) TMT age	1.76	0.03	0.17	–0.07	1								
(5) Product innovation performance	−	0.19	0.35***	0.09	0.20*	*(0.94)*							
(6) Ownership	1.20	0.02	–0.08	−0.34***	0.08	–0.14	1						
(7) Industry	1.08	–0.16	−0.20*	–0.15	–0.04	–0.07	0.02	1					
(8) CEO age	2.25	–0.12	–0.03	–0.13	0.35***	0.13	0.16	0.07	1				
(9) CEO gender	1.08	0.08	0.04	0.17	–0.10	–0.05	–0.13	–0.09	–0.16	1			
(10) CEO tenure	2.56	–0.03	0.06	–0.08	0.15	0.10	0.06	0.11	0.61***	–0.05	1		
(11) TMT tenure	2.84	–0.04	0.03	–0.10	0.46***	0.14	–0.02	0.08	0.20*	0.00	0.50***	1	
(12) TMT working with leader	2.08	–0.01	0.15	–0.17	0.45***	0.24*	0.01	0.04	0.24*	–0.07	0.32**	0.67***	1
Mean	−	4.02	4.24	3.79	38.93	3.84	0.92	1.28	44.36	0.20	12.65	8.04	6.38
SD	−	0.77	0.48	0.61	6.08	0.76	0.27	0.47	7.97	0.40	7.19	4.66	3.45

N = 105; ***p ≤ 0.001, **p ≤ 0.01, *p ≤ 0.05. Cronbach’s alpha appears along the diagonal in the brackets.

To further test the discriminant validity of the key variables, we adopted Fornell and Larcker (1981) criteria. The AVE values for CEO relational leadership, TMT voice behavior, and product innovation performance were 0.77, 0.75, and 0.80, respectively, all above the 0.50 threshold. The square roots of these AVE values belonging to the variables were also much larger than the correlation between them. In addition, all of the loadings of indicators were significant at *p* < 0.01, with standardized loadings ranging from 0.68 to 0.92, providing support for discriminant validity.

### Tests of Hypotheses

We tested our hypotheses using the SPSS PROCESS macro developed by Preacher and Hayes (2008) with 5,000 bootstrap samples. To avoid multicollinearity in the moderation analysis, the independent variable (CEO relational leadership), the moderators (TMT members’ education and TMT members’ age) were mean-centered (Aiken and West, 1991).

The results of the hypothesis tests are summarized in Table 2. CEO relational leadership was positively related to TMT voice behavior (Model 1: *B* = 0.26, SE = 0.06, *p* < 0.001, 95% CI = [0.15, 0.40]), supporting Hypothesis 1. TMT voice behavior had a positive and significant effect on product innovation performance (Model 5: *B* = 0.47, SE = 0.17, *p* < 0.01, 95% CI = [0.13, 0.80]), supporting Hypothesis 2. The results also show that the indirect effect of TMT voice behavior on the relationship between CEO relational leadership and product innovation performance was significant and positive (*B* = 0.12, SE = 0.05, *p* < 0.05, 95% CI = [0.03, 0.31]), supporting Hypothesis 3.

**TABLE 2:** Results of hypothesis tests.

	TMT voice behavior						Product innovation performance			
	Model 1		Model 2		Model 3		Model 4		Model 4	
	*B*	*SE*	*B*	*SE*	*B*	*SE*	*B*	SE	*B*	*SE*
Constant	3.51***	0.42	4.54***	0.32	4.85***	0.35	4.76***	0.35	1.08	0.91
Ownership	–0.16	0.16	–0.05	0.16	–0.15	0.16	–0.02	0.16	–0.41	0.27
Industry	–0.14	0.09	–0.11	0.09	–0.09	0.09	–0.07	0.09	–0.02	0.15
CEO age	–0.00	0.01	–0.01	0.01	–0.01	0.01	−0.01^+^	0.01	0.02	0.01
CEO gender	–0.01	0.11	–0.06	0.10	0.02	0.10	–0.02	0.10	–0.10	0.18
CEO tenure	0.01	0.01	0.01	0.01	0.02^+^	0.01	0.02^+^	0.01	–0.01	0.01
TMT tenure	–0.02	0.01	–0.01	0.01	−0.03*	0.01	−0.03*	0.01	0.01	0.02
TMT working with leader	0.03^+^	0.02	0.03^+^	0.02	0.03^+^	0.02	0.03^+^	0.02	0.03	0.03
Relational leadership	0.26***	0.06	0.25***	0.05	0.27***	0.06	0.25***	0.05	0.08	0.10
TMT education			0.18*	0.08			0.20**	0.07		
RL*TMT education			0.21**	0.08			0.15^+^	0.08		
TMT age					0.02*	0.01	0.02*	0.01		
RL*TMT age					−0.02*	0.01	−0.02^+^	0.01		
TMT voice behavior									0.47**	0.17
*R* ^2^	0.26		0.33		0.32		0.38		0.20	
*F*	4.11***		4.65***		4.34***		4.67***		2.59*	

							**Estimate**		***SE***	**95% C.I.**

Total effect of Relational leadership - Product innovation performance							0.20*		0.10	[0.01, 0.39]
Relational leadership - TMT voice behavior - Product innovation performance							0.12*		0.07	[0.03, 0.31]

N = 105; Bootstrap samples = 5000; ***p < 0.01, **p < 0.01, *p < 0.05, +p < 0.10.

Hypothesis 4a predicted that TMT educational level positively moderates the relationship between CEO relational leadership and TMT voice behavior, and Hypothesis 4b predicted that TMT age negatively moderates the same relationship. Table 2 shows that the interaction term of CEO relational leadership with TMT educational level was significantly and positively associated with TMT voice behavior (Model 2: *B* = 0.21, SE = 0.08, *p* < 0.01, 95% CI = [0.06, 0.36]), and the interaction term of CEO relational leadership with TMT age was significantly and negatively associated with TMT voice behavior (Model 3: *B* = –0.02, SE = 0.01, *p* < 0.05, 95% CI = [–0.05, –0.01]). These findings support Hypotheses 4a and 4b.

**FIGURE 2:**
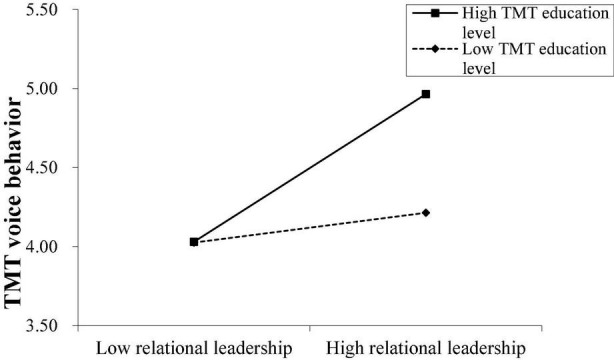
The moderating role of TMT educational level.

**FIGURE 3:**
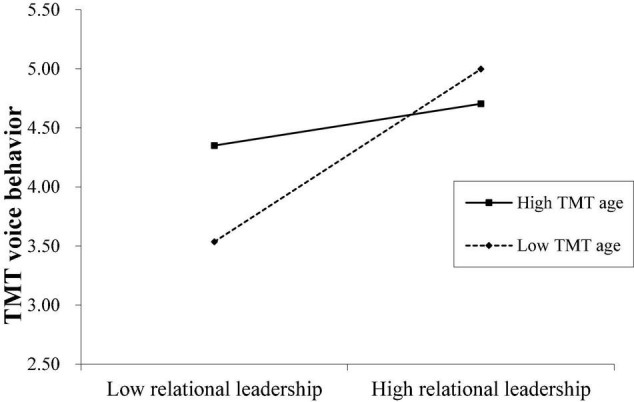
The moderating role of TMT age.

To determine the nature of the moderating effect, we plotted the interaction by computing slopes one standard deviation above and below the mean of the moderators. Figure 2 shows that for TMTs with a high level of education, the positive relationship between CEO relational leadership and TMT voice behavior was stronger (simple slope = 0.47, *p* < 0.00) compared to TMTs with a lower level of education (simple slope = 0.09, *p* = 0.29, n.s.). Figure 3 shows that for younger TMTs, the positive relationship between CEO relational leadership and TMT voice behavior was stronger (simple slope = 0.49, *p* < 0.00) compared to older TMTs (simple slope = 0.12, *p* = 0.16, n.s.).

## Discussion

This study examines whether, how, and when CEO relational leadership leads to product innovation performance. Based on upper echelons theory and relational leadership literature, this study finds that CEO relational leadership is positively related to TMT voice behavior, which in turn promotes product innovation performance. The results also reveal that when TMT educational level is higher and TMT members are younger, CEO relational leadership is more likely to motivate TMT members to voice their opinions, which in turn improves product innovation performance.

### Theoretical Implications

Our findings contribute to the existing literature in several ways. First, this study extends the literature on CEO leadership and firm innovation. Although previous studies have examined the effects of various types of CEO leadership on firm innovation, most have focused on the personal attributes of CEOs and have largely ignored the relational dynamics and process of leadership (Reitz, 2015; McCauley and Palus, 2021). A growing body of leadership research has explored leadership influence from a relational perspective (Uhl-Bien, 2006). This study, to our knowledge, is the first to empirically examine the relationship between CEO relational leadership and product innovation performance. The findings suggest that by emphasizing relationships and relational interaction within an organization, CEO relational leadership can promote communication and understanding among TMT members and motivate them to voice their concerns and suggestions, which translates to improved product innovation performance. This study demonstrates that CEOs can shape innovation outcomes not only by modeling transformative behavior (Kraft and Bausch, 2016) or building an innovative vision (Caridi-Zahavi et al., 2016), but also by engaging in trivial daily conversations and relational interaction with TMT members (Uhl-Bien, 2006; Cunliffe and Eriksen, 2011). Thus, relational leadership provides a nuanced way to understand how CEO leadership facilitates product innovation.

Second, this study complements research on how CEO leadership results in firm outcomes and enriches the CEO–TMT interface literature. Although the effect of CEO leadership on firm outcomes has been widely examined, research on the complex nature of this relationship is relatively scarce (Ling et al., 2008; Chen et al., 2014). Upper echelons research states that a CEO’s effect on firm outcomes can be transmitted by TMT dynamics, and the interplay between CEO and TMT can result in a stronger explanation of the heterogeneity of firms’ outcomes (Carpenter et al., 2004; Liu et al., 2018). In this study, we introduce TMT voice behavior as the interpersonal dynamic in TMT and verify its positive influence on product innovation performance. The intervening mechanism helps us have a better understanding of how CEO relational leadership influences product innovation performance. Specifically, CEO relational leadership, by engaging TMT members in rich dialog, encourages TMT members to voice their opinions, which results in deeper interaction between CEOs and TMT members and improves product innovation performance. The interplay between CEO relational leadership and TMT voice provides a strong explanation of how top executives influence firm outcomes.

Third, this study contributes to research on the linkage between CEO leadership and TMT voice behavior by identifying two conditional factors. Although Cortes and Herrmann (2020) have demonstrated that transformational CEOs have a positive impact on employee participation, they do not explore the boundary conditions that may place limits on how CEO leadership actually affects employees’ willingness to voice their ideas. We move the research forward by introducing TMT educational level and TMT age as boundary conditions. TMT educational level and TMT age, as the prominent demographic details that reflect TMT members’ values and cognition, affect their interpretation of, and reaction to, external stimuli (Hambrick and Mason, 1984). The results demonstrate that TMT education and age significantly moderate the relationship between CEO relational leadership and TMT voice behavior. TMTs who are younger and more educated are more likely to respond to relational CEOs and voice their suggestions and ideas. In this manner, this study enriches the theoretical model of when CEO leadership facilitates TMT or employee voice behavior, which then advances product innovation performance.

Finally, by examining the effect of CEO relational leadership on product innovation performance, we also enrich the literature on relational leadership. Although books, reviews, and theoretical papers have increasingly highlighted the importance of relational leadership (Uhl-Bien, 2006; Uhl-Bien and Ospina, 2012), empirical works are still scarce. Studies on sustainable development and regional energy activities in environmental science (Kurucz et al., 2017; Ruppert-Winkel, 2018; Nicholson and Kurucz, 2019), decision-making in healthcare (Fulop and Mark, 2013), and strategic decision quality in top management teams (Carmeli et al., 2012) are too scattered across academic fields to generate substantive knowledge on relational leadership. Ours is one of the few studies to examine the firm-level consequences of relational leadership; it paves the way for future empirical research to explore whether relational leadership causes certain outcomes in organizations.

### Practical Implications

Our findings also have significant implications for practitioners. Accelerating technological change and intensified competition make rapid and successful product innovation a must for firms’ sustainable competitiveness (Chen et al., 2014). However, innovation cannot be achieved by any single member of an organization. It is a collective effort that requires every member work with others to solve problems together. Therefore, it is important for leaders in a firm to promote connections and interactions among organization members. Relational leadership is highly relevant to these practices. This study provides evidence that CEO relational leadership is beneficial for product innovation performance via TMT voice behavior. Thus, to facilitate product innovation, CEOs should develop relational skills and pay attention to daily interaction and communication. More specifically, CEOs should initiate conversations with TMT members and employees in formal or informal ways rather than waiting to receive reports from their employees. This is especially necessary in the digital era, as organizations become more decentralized and employees become partners and collaborators who wish to be valued.

Additionally, the mediating mechanism of TMT voice behavior suggests that CEOs should encourage TMT members to voice their opinions by relating to and interacting with them. Relational leaders can also create an environment of open communication to encourage employees to express their concerns and suggestions. Lastly, our study demonstrates that TMT educational level and TMT age are important boundary conditions in the relationship between CEO leadership and TMT voice behavior. Younger, educated executives are more open to new ideas and have the ability to deal with complicated situations (Barker, and Mueller, 2002). They are more likely to engage in positive relationships with relational CEOs and express their innovative ideas and suggestions to bring about change. This finding suggests that apart from developing relational skills, CEOs should also be careful in the selection and appointment of TMT members. Irrespective of their size, firms that emphasize innovation should select TMT members who are not only technically competent, but also willing to express their opinions to contribute to the firm’s development.

### Limitations and Future Research

This study has several limitations that are relevant for future research. First, it identifies TMT educational level and age as moderators, as these two demographic characteristics represent different cognitive capabilities. However, we did not directly consider TMT members’ psychological traits and personality. Although the use of proxy variables is a common practice of upper echelons research, the recently systematic reviews of this theory (Abatecola and Cristofaro, 2020; Neely Lovelace et al., 2020) have pointed out the defect of using demographic characteristics as the proxies of cognition and psychological traits. The proxy variables may not capture actual psychological traits and personality of TMT members. While TMT members with different personality may react differently to the behavior of relational CEOs which affects their willingness to speak up, as LePine and Van Dyne (2001) found that people with personality traits of conscientiousness, extraversion and agreeableness are more likely to voice. Therefore, one potentially interesting extension of this study would be to explore the interactive effect of CEO relational leadership and TMT personality or psychological traits on TMT voice behavior by directly measuring these psychological constructs.

Second, the consequences of CEO relational leadership warrant more empirical examination. In this study, we examined the effect of CEO relational leadership on product innovation performance. CEO relational leadership may lead to various outcomes that warrant more research, such as inter-firm collaboration at the organizational level, and organizational loyalty at the individual level.

Third, we collected data from 105 Chinese SMEs and studied the effect of CEO relational leadership only in the Chinese context. China is considered a relational society (Ren et al., 2019), so it provides an appropriate context to test the effect of relational leadership. However, focusing on China may limit the generalizability of the findings. The influence of relational leadership on TMT voice behavior may differ in different cultures. An extension of this study is to empirically test whether and how the effect of relational leadership differs in western and eastern cultures.

Finally, we initially contacted CEOs who we were familiar with and then asked them to recommend other SMEs. This non-random sampling may lead to sampling selection bias. Future research is recommended to use random sampling methods to validate the findings.

## Data Availability Statement

The raw data supporting the conclusions of this article will be made available by the authors, without undue reservation.

## Author Contributions

All authors listed have made a substantial, direct, and intellectual contribution to the work and approved it for publication.

## Conflict of Interest

The authors declare that the research was conducted in the absence of any commercial or financial relationships that could be construed as a potential conflict of interest.

## Publisher’s Note

All claims expressed in this article are solely those of the authors and do not necessarily represent those of their affiliated organizations, or those of the publisher, the editors and the reviewers. Any product that may be evaluated in this article, or claim that may be made by its manufacturer, is not guaranteed or endorsed by the publisher.
